# Reactive Oxygen Species, Superoxide Dimutases, and PTEN-p53-AKT-MDM2 Signaling Loop Network in Mesenchymal Stem/Stromal Cells Regulation

**DOI:** 10.3390/cells7050036

**Published:** 2018-05-01

**Authors:** Satoru Matsuda, Yukie Nakagawa, Yasuko Kitagishi, Atsuko Nakanishi, Toshiyuki Murai

**Affiliations:** 1 Department of Food Science and Nutrition, Nara Women’s University, Kita-Uoya Nishimachi, Nara 630-8506, Japan; yukiie0028@yahoo.co.jp (Y.N.); y_kitagishi@live.jp (Y.K.); 2 Department of Food and Nutrition, Faculty of Contemporary Human Life Science, Tezukayama University, Nara 631-8501, Japan; gah00635@nifty.com; 3 Department of Microbiology and Immunology, Graduate School of Medicine, Osaka University, Suita 565-0871, Japan; pi3kp10@outlook.jp

**Keywords:** p53, PTEN, AKT, MDM2, superoxide dismutase, SOD, reactive oxygen species, ROS, mesenchymal stromal/stem cell, MSC, stemness

## Abstract

Mesenchymal stromal/stem cells (MSCs) are multipotent cells that can differentiate to various specialized cells, which have the potential capacity to differentiate properly and accelerate recovery in damaged sites of the body. This stem cell technology has become the fundamental element in regenerative medicine. As reactive oxygen species (ROS) have been reported to adversely influence stem cell properties, it is imperative to attenuate the extent of ROS to the promising protective approach with MSCs’ regenerative therapy. Oxidative stress also affects the culture expansion and longevity of MSCs. Therefore, there is great need to identify a method to prevent oxidative stress and replicative senescence in MSCs. Phosphatase and tensin homologue deleted on chromosome 10/Protein kinase B, PKB (PTEN/AKT) and the tumor suppressor p53 pathway have been proven to play a pivotal role in regulating cell apoptosis by regulating the oxidative stress and/or ROS quenching. In this review, we summarize the current research and our view of how PTEN/AKT and p53 with their partners transduce signals downstream, and what the implications are for MSCs’ biology.

## 1. Introduction

Mesenchymal stromal/stem cells (MSCs) are multipotent stem cells that are present in almost all fetal and adult issues, which are characterized by their ability to differentiate into several specialized cells [1]. MSCs are progenitors of the various important cells that have emerged as vital tools for medical engineering. MSCs have been isolated from a number of different tissues such as cord blood, placenta, and bone marrow, which are being investigated for several tissue repair, immune modulation, and so on [2]. For example, studies have reported the transplantation of MSCs from bone marrow as a strategy for cardiac repair following myocardial infarction [3]. Enhancing the viability of implanted MSCs and restoring cellular mechanisms are therefore serious in order to gain adequate outcomes with the therapy. Reactive oxygen species (ROS) and nonspecific inflammation have been hypothesized to lead to the loss of the transplanted MSCs from concerned sites [4]. At the inflammation sites, excessive immune cells would accumulate, and they could produce ROS. Furthermore, aging and senescence usually reduce MSCs’ expansion [5], which is also critical for their clinical applications. MSCs easily undergo replicative senescence, limiting the number of divisions, and proliferation exaggeratedly decreases. In general, aging and/or senescence is associated with an excess of oxidative stress, which could limit the quality of MSCs [6]. On the other hand, evidence has suggested that the regulation of mitochondria dynamics is indispensable for the fruitful differentiation of MSCs [7]. These findings have important implications in MSCs’ biology, which requires a greater understanding of the contributions of oxidative stress and/or ROS to the MSCs. In particular, there is a prerequisite to identify the methods to manipulate MSCs to reduce ROS in both the MSCs themselves and in their tissue microenvironment in order to improve the potential and/or the quality of MSCs. Further study of the oxidative stress in MSCs is also imperative in order to precisely estimate the roles, risks, and mechanisms for the longevity of MSCs.

## 2. ROS Is Involved in the Differentiation and Senescence of MSCs

**Figure 1:**
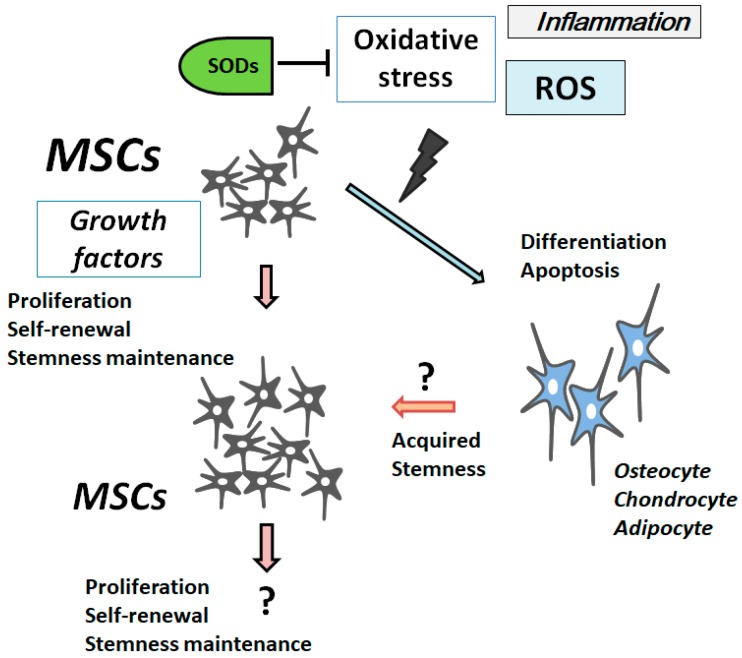
Illustration of mesenchymal stromal/stem cells (MSCs) proliferation and/or differentiation in response to the extracellular growth factor stimulation and/or oxidative stress. The model shows that several triggers including oxidative stress, superoxide dismutases (SODs), reactive oxygen species (ROS), inflammation, and growth factors could affect MSCs and their destinations. SODs reduce some of the oxidative stress by dismuting superoxide. Note that some critical routes have been omitted for clarity.

Generally, ROS inhibit MSCs’ proliferation, increase the senescence, and enhance several cell differentiations (Figure 1). The ROS represent a group of oxygen-containing minor molecules, which react freely with various chemical structures. Examples of ROS include peroxides, superoxides, hydroxyl radicals, and singlet oxygen. ROS have been considered to bring cellular dysfunction and cell death/apoptosis via the damaging oxidation of cellular components. Now, it has been revealed ROS have important roles in normal cell physiology in addition to the pathology of several diseases such as tissue degenerative disorder and cancer [8]. Whereas unregulated levels of ROS may be harmful, under physiological conditions, a regulated basal level of ROS is even necessary in order for them to assist as second messengers for regulatory functions, and they are also advantageous for the maintenance of cellular functions [9]. The impacts of ROS on MSCs’ differentiation and/or proliferation have attracted a deal of interest due to potential applications in medical therapeutics.

Oxidative stress, ROS, and senescence/aging may enhance cell differentiation (Figure 1). For example, the differentiation of chondrocyte is accelerated by an oxidative stress, and chondrogenesis is inhibited by the administration of antioxidants [10]. On the contrary, the upregulation of ROS inhibits the osteogenic differentiation of MSCs [11]. Whereas a low level of ROS could promote osteogenesis, high levels of ROS could promote MSCs to adipocytes [12]. Antioxidant enzymes such as superoxide dismutase (SOD) and catalase have been shown upregulated upon the osteogenic differentiation, which led to a dramatic decrease in the intracellular ROS level [13]. Antioxidant enzymes are also upregulated during adipogenesis differentiation in MSCs [14]. Consistent with this, it has been shown that the ROS scavenger *N*-acetylcysteine (NAC) inhibits adipogenesis in stromal cells [15]. The NAC improves antioxidant capacity by augmenting glutathione levels, which also inhibits some of the ROS levels. Increasing ROS levels may stimulate chondrocyte hypertrophy, which is inhibited by the NAC [16]. Conceivably, MSCs may rely on oxidative mitochondrial metabolism, and mitochondrial metabolism may be important for the MSCs’ differentiation [17]. It has been demonstrated that ROS generated by mitochondria are imperious for the activation of adipogenic transcription factors [18]. Similarly, mitochondrial biogenesis and oxygen intake upsurge meaningfully during adipogenesis [19]. Consistently, inhibiting mitochondrial respiration significantly suppresses several cell differentiations, reducing adjacent ROS [20].

Cellular ROS metabolism might be firmly controlled by a variety of molecules involved in redox machinery. However, less is known about the initial mechanism and/or regulation of signaling molecules by ROS. One mechanism by which ROS are thought to exert their effects may be through the flexible regulation of target molecules such as PI3K and AKT [21]. For example, high-density lipoprotein (HDL) has been described as possessing a variety of functional properties in addition to the ability to transport cholesterol [22]. HDL has also been shown to eliminate the effects of ROS in vascular walls [23]. To reduce ROS, HDL has been shown to protect endothelial cells from cell apoptosis [24]. Similarly, HDL protects MSCs from apoptosis by activation of the PI3K/AKT pathway [25,26]. Furthermore, the HDL has promoted MSCs’ survival and function [25,26], indicating the importance of the PI3K/AKT pathway.

## 3. Characterization of Superoxide Dismutases

A group of metal-containing enzymes named superoxide dismutases (SODs) have vigorous antioxidant roles categorized by their scavenging of ROS [27]. The SODs have been thought to be the first line of protection arrangement against oxidative stresses, which implies that SODs can play an important defensive role in several cell apoptosis. Before some of ROS can oxidize critical DNA and/or proteins, SODs catalyze the reaction of superoxides to the less damaging/reactive hydrogen peroxide [28]. The presence of metals such as Cu, Zn, Mn, or Fe may be essential for this function in the system [29]. So, altered metal homeostasis in cells may be the cause of endogenous oxidative stress [30]. Three types of SODs are known in mammalian species. The most abundant cytosolic enzyme is SOD1. The loss of SOD1 increases the total level of ROS, which is thought to trigger oxidative DNA damage to cells. It has been shown SOD1-null animals develop some age-related diseases [31]. Amazingly, SOD1 may upturn the therapeutic potential of MSCs [32]. SOD2 is located in the mitochondrial matrix [33], which is the critical site of free radical production from the electron transportation chain. SOD2 is required for maintaining mitochondrial functions and reliability [34]. One of the primary functions of SOD2 might be to protect mitochondrial DNA against oxidative damage [35]. The SOD2 gene is subjected to regulation by a number of inflammatory cytokines and growth factors [36]. It has been shown that SOD2 overexpression causes an increase in ATP production through energetic mitochondrial respiration [37]. The induction of osteogenesis in MSCs is associated with an upregulation of SOD2 and a decrease in ROS levels [38]. Additionally, the reduction of SOD2 diminishes the expression of an adipogenesis marker, which results in higher ROS production [39]. SOD3 is secreted to the extracellular matrix in cells and tissues [40]. Downregulation of SOD3 has been shown to lead DNA copy number change and/or hypermethylation in the promoter region of genes [41]. It has been revealed that overexpressed SOD3 causes hypoxic accumulation of hypoxia inducible factor-1α (HIF1α) in cells [42]. There is an increase in SOD3 expression with the differentiation of MSCs into adipocytes [43]. In addition, SOD3 levels have been shown to decrease upon chondrogenesis [43].

## 4. PI3K/AKT/PTEN and p53 Signaling Are Involved in Maintenance of MSCs’ Proliferation and Stemness

**Figure 2:**
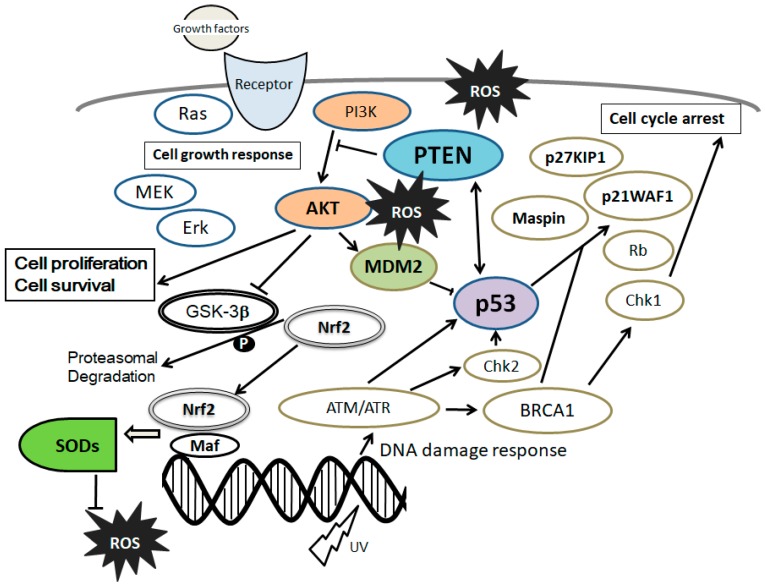
Schematic representation of the integrative model of tumor suppressor molecules signaling, including phosphatase and tensin homolog deleted on chromosome 10 (PTEN) and p53 in response to the extracellular growth factor stimulation and/or oxidative stress. Typical examples of molecules known to act on the DNA damage response and cell proliferation or cell cycle progression via the regulatory intracellular pathway are shown. Note that some critical signaling has been omitted for clarity.

In MSCs, excess ROS can impair self-renewal, differentiation capacity, and proliferation [44]. Concordantly, antioxidants stimulate MSCs’ proliferation [45]. The MSCs’ stemness is maintained by inhibiting cellular senescence through a PI3K/AKT pathway [46]. Moreover, the PI3K/AKT pathway has been shown to be involved in maintaining embryonic stem cell pluripotency [47]. Activation of the PI3K/AKT signaling may have dynamic roles in maintaining the pluripotency of stem cells [48], which is also involved in enhanced cell proliferation [49] (Figure 2). In addition, it has been reported that PI3K/AKT is associated with the regulation of stem cell fate [50]. Stromal cell-derived factor 1 (SDF1) is an important chemokine in stem cell mobilization, and plays a critical role in the biological functions of MSCs by enhancing PI3K expression [51]. Muc1 is a member of the carbohydrate-binding protein family that contributes to MSCs’ survival and stemness via the PI3K/AKT signaling [52].

Studies have shown an antioxidant role for tumor suppressor molecules: they adjust the expression of some antioxidant genes in response to oxidative stress [53]. For example, PTEN (phosphatase and tensin homolog deleted on chromosome 10) performs as this, which is a tumor suppressor gene that is often deleted and/or mutated in a lot of human cancers [54]. PTEN depressingly regulates the activity of PI3K/AKT signaling through converting PIP3 into PIP2. The PTEN gene can be upregulated by p53 and PPAR*γ*, while NF-*κ*B and TGF-*β* downregulate *PTEN* expression [55,56]. Interestingly, some components in rosemary extract can repress PTEN expression in culture cells [57]. The related PI3K/AKT/PTEN pathway signaling takes place as a pivotal determinant of cell fate regarding senescence and apoptosis, which is mediated by ROS generation [58]. The signaling protects cells against oxidative damage partially via a transcription factor Nrf2 activation [59]. The Nrf2 forms a heterodimer with other transcription factors such as Maf protein after translocation into the nucleus, which in turn binds to the regulatory sequence termed antioxidant response elements (ARE) [60]. ARE is located at the promoter region of certain genes encoding several antioxidant enzymes [60]. Silencing of Nrf2 considerably inhibits the expression of SOD1 and/or SOD2 [61]. In addition, the Nrf2-Maf complex level is significantly increased by oxidative stress [61,62]. Then, AKT-mediated Nrf2-Maf activation meaningfully attenuates oxidative stress and cellar apoptosis [61,62] (Figure 2).

The NRF2 plays a key role in the conservation of MSCs’ self-renewal and differentiation potential by regulating p53 [62]. Additionally, p53 is implicated in protecting cells from the attack of oxidative stress [63]. It has been shown that pretreatment with curcumin, which is a phytochemical substance found in turmeric spice, noticeably enhances the anti-apoptotic ability of stem cells [64], and they can preserve their viability by the inhibition of PTEN and p53 signaling and/or activation of AKT and HO-1 signaling [65]. In addition, the antioxidant curcumin has been reported to modulate this PTEN/AKT/p53 axis to exhibit its cell protective effects. Moreover, curcumin induces kinds of senescence, which are indicated by elevating the expression of senescence markers [66]. It has also been suggested that nuclear PTEN induced by ATM-mediated phosphorylation plays a unique role to protect cells upon oxidative damage [67,68] (Figure 2).

## 5. Involvement of PTEN-p53-AKT-MDM2 Loop in MSCs Regulation

It has been proposed that low levels of p53 induce cell cycle arrest, whereas high levels of p53 induce apoptosis [69]. The PI3K/AKT activation runs into the inhibition of p53 by activating another tumor suppressor, MDM2 [70]. MDM2 is an oncoprotein that regulates tumorigenesis, whose mRNA level is regulated by p53 in response to oxidative stress and/or DNA damage [71]. Subcellular localization of the MDM2 is post-translationally modulated by PI3K/AKT [72]. Consequently, PI3K/AKT and p53 affect the process of apoptosis in opposed ways. In addition, there are cross-talks between AKT and p53 involving transcription as well as post-translational modifications [72,73]. Moreover, the subsequent p53-induced expression of PTEN causes the p53–PTEN interaction, which suppresses the cell survival through PI3K/AKT signaling [70]. PTEN associates with p53 and regulates the transcriptional activity of p53 by modulating its DNA binding [74]. AKT kinase phosphorylates MDM2 to translocate into the nucleus, as mentioned formerly. In addition, PTEN is required for the maintenance of p53 acetylation, which is required for target gene transcription [75]. PTEN has also been shown to interact with p53 and prevent its degradation. The p53 and MDM2 complex transports from the nucleus into the cytoplasm, where MDM2 serves as an E3 ubiquitin ligase [76]. Attenuation of the PI3K/AKT pathway by PTEN protects p53 from MDM2-mediated degradation and inactivation. The levels of p53 could be positively related to the amount of oxidative DNA damage.

**Figure 3:**
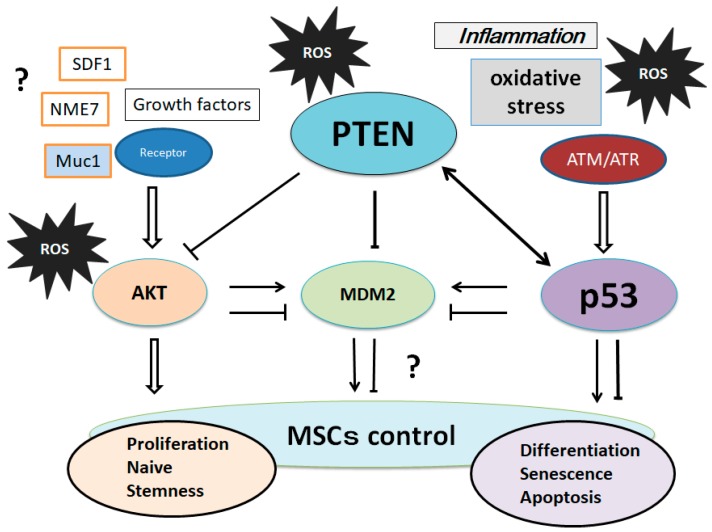
Suggestion of various molecular regulatory loops involving the PTEN-p53-AKT-MDM2 network on the controls of MSCs is shown. Interactions are shown as arrows to mean activation, while hammerheads mean inhibition. Expression of these molecules is regulated by genetic, epigenetic, and transcriptional changes, which may result in the MSCs’ regulation. Note that some critical pathways have been omitted for clarity.

On the other hand, AKT activation can overcome both the p53-independent cell cycle checkpoint and apoptosis that is induced by the oxidative DNA damage. The PTEN-p53-MDM2-AKT loop in MSCs’ regulation now may become dominant (Figure 3). As mentioned above, PTEN and p53 are known to interact and regulate each other, which could be at the important control machinery for switching between survival and death. These cross-talks are frequently a combination of mutually antagonistic pathways, which may frequently serve as an added regulatory effect on the expression of key genes involved in MSCs. Interestingly enough, soy isoflavone genistein induces an autoregulatory loop between PTEN and p53 [77]. The induction of PTEN expression and nuclear accumulation by genistein elicits a sequence of PTEN-dependent increased nuclear p53 accumulation, enhanced PTEN/p53 physical interaction, and increased recruitment of the PTEN/p53 complex to the p53 binding sites of the PTEN promoter [77,78]. Eventually, it has been informed that genistein suppresses the adipogenic differentiation of MSCs in a dose-dependent manner [79].

It has been shown that zinc deficiency modulates the PTEN-p53-MDM2-AKT signaling axis [80]. (Figure 3) In addition, the enhanced proliferation potential of MSCs has been accompanied by the upregulation of multiple genes, including AKT and Mdm2 [81]. Many dietary compounds are known to have health benefits owing to their antioxidative and anti-inflammatory properties. Among them, the polyphenols from blueberries have been involved in the ultraviolet-activated p53/MDM2 DNA repair system by restoring the cell membrane potential [82]. In addition, curcumin downregulates MDM2 and upregulates p53 [83], which exhibits cell protective effects. Chitosan is a deacetylated polysaccharide derivative of chitin that is contained in the shells of crustacean such as crabs and shrimps in nature, which activates the PI3K/AKT pathway [84], and has also been shown to enhance mineral deposition during the osteogenic differentiation of MSCs [85]. At these points, again, the PTEN-p53-MDM2-AKT loop becomes dominant in MSCs’ biology (Figure 3). The microenvironment of cells/tissues could be driven by these diet ingredients.

## 6. Future Perspectives

MSCs may have huge therapeutic potential, however, oxidative stress damages on MSCs may be leading to problems. Therefore, there is unlimited need to identify novel methods to optimize ROS levels in MSCs to enhance their regenerative abilities, so that their full therapeutic potential can be realized. Key molecules may be regulated and interact each other at multiple levels including transcription, protein modulation, and protein stability, and so forth. Understanding the regulation is also crucial for the effective design of novel MSCs’ therapeutics. Numerous interactions may support the biological plausibility that the combination of variants of the PTEN-p53-AKT-MDM2 network could result in more comprehensive treatment. It will be important to understand the mechanism-of-action, which will lay a foundation for targeting SODs in the appropriate stages of MSCs.

Human diet usually consists of complex combinations of lipids and/or nutrients that might act synergistically or antagonistically. Further mechanistic studies are needed in order to understand the precise molecular mechanisms for the effective diet with their alteration. Looking forward, precisely understanding these regulations is crucial for MSCs’ therapeutic intervention. Especially, long-term studies are obligatory in order to explore combinations with other therapies that are known to reduce ROS levels.

## Abbreviations

ARE	antioxidant response elements
ATF2	activating transcription factor 2
ATP	adenosine triphosphate
DNA	deoxyribonucleic acid
GSK3β	glycogen synthase kinase-3β
HIF1α	hypoxia inducible factor-1α
Maf	macrophage activating factor
MAPK	Mitogen-activated Protein Kinase
MSCs	Mesenchymal stromal/stem cells
NAC	*N*-acetylcysteine
Nrf2	NF-E2-related factor-2
PIP3	phosphatidylinositol 3,4,5-triphosphate
PIP2	phosphatidylinositol 4,5-bisphosphate
PI3K	phosphoinositide-3 kinase
PKC	protein kinase c
PPAR	peroxisome proliferator-activated receptor
PPREs	PPAR response elements
PTEN	phosphatase and tensin homologue deleted on chromosome 10
PUFAs	polyunsaturated fatty acids
ROS	reactive oxygen species
SDF1	stromal cell-derived factor 1
SODs	superoxide dismutases
